# Changes in the Serum Metabolome of Patients Treated With Broad-Spectrum Antibiotics

**DOI:** 10.20411/pai.v5i1.394

**Published:** 2020-12-29

**Authors:** George E. Jaskiw, Mark E. Obrenovich, Sirisha Kundrapu, Curtis J. Donskey

**Affiliations:** 1 Psychiatry Service, Veterans Affairs Northeast Ohio Healthcare System (VANEOHS), Cleveland, Ohio; 2 School of Medicine, Case Western Reserve University, Cleveland, Ohio; 3 Pathology and Laboratory Medicine Service, VANEOHS, Cleveland, Ohio; 4 Research Service, VANEOHS, Cleveland, Ohio; 5 Department of Chemistry, Case Western Reserve University, Cleveland, Ohio; 6 Geriatric Research, Education and Clinical Center, VANEOHS, Cleveland, Ohio

**Keywords:** serum, metabolome, antibiotic, L-tyrosine, L-phenylalanine, L-tryptophan, *Clostridia*

## Abstract

**Background:**

The gut microbiome (GMB) generates numerous small chemicals that can be absorbed by the host and variously biotransformed, incorporated, or excreted. The resulting metabolome can provide information about the state of the GMB, of the host, and of their relationship. Exploiting this information in the service of biomarker development is contingent on knowing the GMB-sensitivity of the individual chemicals comprising the metabolome. In this regard, human studies have lagged far behind animal studies. Accordingly, we tested the hypothesis that serum levels of chemicals unequivocally demonstrated to be GMB-sensitive in rodent models would also be affected in a clinical patient sample treated with broad spectrum antibiotics.

**Methods:**

We collected serum samples from 20 hospitalized patients before, during, and after treatment with broad-spectrum antibiotics. We also collected samples from 5 control patients admitted to the hospital but not prescribed antibiotics. We submitted the samples for a non-targeted metabolomic analysis and then focused on chemicals known to be affected both by germ-free status and by antibiotic treatment in the mouse and/or rat.

**Results:**

Putative identification was obtained for 499 chemicals in human serum. An aggregate analysis did not show any time x treatment interactions. However, our literature search identified 10 serum chemicals affected both by germ-free status and antibiotic treatment in the mouse or rat. Six of those chemicals were measured in our patient samples and additionally met criteria for inclusion in a focused analysis. Serum levels of 5 chemicals (p-cresol sulfate, phenol sulfate, hippurate, indole propionate, and indoxyl sulfate) declined significantly in our group of antibiotic-treated patients but did not change in our patient control group.

**Conclusions:**

Broad-spectrum antibiotic treatment in patients lowered serum levels of selected chemicals previously demonstrated to be GMB-sensitive in rodent models. Interestingly, all those chemicals are known to be uremic solutes that can be derived from aromatic amino acids (L-phenylalanine, L-tyrosine, or L-tryptophan) by anaerobic bacteria, particularly *Clostridial* species. We conclude that judiciously selected serum chemicals can reliably detect antibiotic-induced suppression of the GMB in man and thus facilitate further metabolome-based biomarker development.

## INTRODUCTION

The gut microbiome (GMB) generates numerous small (< 2,000 Da) chemicals that can be absorbed, biotransformed, and either incorporated or excreted by the host [1–3]. The levels of such chemicals, their relationships, and temporal patterns can offer information about the state of the GMB, the gut wall, the liver, or other participating systems [4, 5]. GMB-derived metabolites may themselves be bioactive [6]. Thus, the characterization of their chemical profiles may have a wide range of diagnostic, prognostic, or therapeutic applications [4, 5].

Development of such potential biomarkers requires identifying the GMB-sensitive constituents of the metabolome. While germ-free mice and rats (GF) have proved invaluable in this regard [7], they have no true human homologue. An alternative approach is to evaluate antibiotic regimens that in rodent models selectively suppress certain microbial taxa [8–11] or eliminate most of the GMB, thus generating a pseudo-GF status [12]. Although it has been more than 65 years since antibiotic treatment was first observed to lower excretion of volatile phenols in man [13], a current literature search identified only 5 studies of antibiotic effects on the human serum metabo-lome [14–18]. Given the high and rising global use of antibiotics [19], the paucity of metabolomic studies in antibiotic-exposed humans constitutes a striking knowledge gap that must be acknowledged, explained, and resolved.

In man, antibiotic exposure is associated with numerous side-effects [20] including suppression of the GMB and weakening of resistance to enteric pathobionts [21, 22]. Gut overgrowth by health-care-associated pathogens such as *Clostridium difficile* and vancomycin-resistant *Enterococci* [11, 23, 24] imposes substantial morbidity, mortality, and economic burden [25]. Such considerations limit human antibiotic studies to clinical samples, thus constraining control over demographic (age [26], socioeconomic [27]), intrinsic (genetic [28]), and other factors (diet [29], physical activity [30], colonic transit time [31], medications [32], morbidity, environment [33]) that can affect the human GMB and/or metabolome [34]. Not surprisingly, many chemicals within the human metabolome show very high inter- and intra-individual variance [3, 35, 36]. Ingestion of a single cup of coffee, for instance, can elevate excretion of certain GMB-derived small phenolic chemicals by several-hundred-fold [37].

The lower variance of chemical levels in serum [36] relative to those in urine [35] or feces [38] should make the human serum metabolome particularly useful for studying antibiotic effects. The small number of such reports [14–18] may be due to both technical and statistical barriers. Identification and quantification of chemicals using a targeted analytic approach requires having authentic standards and generating calibration curves for each analyte of interest. This can be highly resource intensive. Untargeted approaches that draw on spectral libraries provide only semi-quantitative information and putative identification but generate data on thousands of chemicals [39]. To deal with the resulting large numbers of variables, the False Discovery Rate (FDR) [40] is commonly applied. Such a statistical constraint may be excessively strict at times, resulting in type II statistical errors. Given the bias against publishing negative results [41], investigators may be discouraged from conducting investigations of antibiotic effects on the serum metabolome or from publishing ostensibly negative data from completed studies.

A complementary approach would be to *a priori* identify a discrete number of chemicals expected to be consistently detectable as antibiotic-responsive by univariate analysis. Such anchor points would confirm antibiotic effects overall and justify the search for additional sensitive chemicals. Accordingly, we conducted untargeted metabolomic analysis of serum from patients treated with wide-spectrum antibiotic and then subjected the aggregate data to a focused, pre-planned analysis guided by results from animal studies.

## MATERIALS AND METHODS

### Participants

The study protocol was approved by the Institutional Review Board of the Veterans Affairs Northeast Ohio Healthcare System (VA NEOHS). Newly hospitalized patients who were prescribed broad-spectrum antibiotic regimens were recruited for enrollment. Exclusion criteria included systemic antibiotic treatment within the month before admission, altered mental status or dementia, lack of availability of blood serum specimens prior to the start of antibiotic therapy, anticipated length of hospital stay less than 3 days, tube feeds, and end-stage renal or liver disease. Serum specimens were collected prior to the start of antibiotic therapy, on day 2 to 3 of therapy, and 3 to 5 days after completion of antibiotic therapy. To evaluate the potential for large shifts in the serum metabolome during hospitalization in the absence of antibiotic therapy, serum specimens were collected at the same time points for 5 control patients admitted to the hospital but not prescribed antibiotics. These control patients were not matched to the experimental patients based on clinical characteristics. The serum specimens were stored at −80°C. After completion of specimen collection, coded samples were shipped on dry ice to Metabolon© (Morrisville, NC) where they were inventoried and stored at −80°C until analyzed.

### Sample Preparation and Technical Analysis

All sample preparation and analysis was conducted by Metabolon©. In brief, samples were extracted using proprietary methods and the resulting extract divided into 2 fractions, 1 each for analysis on gas chromatography/mass spectroscopy (GC/MS) and liquid chromatography (LC)/MS platforms [42]. The LC/MS portion of the platform was based on a Waters ACQUITY UPLC and a Thermo-Finnigan LTQ mass spectrometer, which consisted of an electrospray ionization source and linear ion-trap mass analyzer. The sample extract was split into 2 aliquots, dried, then reconstituted in acidic or basic LC-compatible solvents, each of which contained 11 or more injection standards at fixed concentrations. One aliquot was analyzed using acidic positive ion optimized conditions and the other using basic negative ion optimized conditions in 2 independent injections using separate dedicated columns. The samples destined for GC/MS analysis were re-dried under vacuum desiccation for a minimum of 24 hours prior to being derivatized under dried nitrogen using bistrimethyl-silyl-triflouroacetamide. The GC column was 5% phenyl and the temperature ramp was from 40° to 300° C in a 16-minute period. Samples were analyzed on a Thermo-Finnigan Trace DSQ fast-scanning single-quadrupole mass spectrometer using electron impact ionization. The instrument was tuned and calibrated for mass resolution and mass accuracy on a daily basis. The information output from the raw data files was automatically extracted. Compounds were identified by comparison to library entries of purified standards or recurrent unknown entities [42] (for details see Supplementary Information, Methods).

### Statistical Analysis

#### General:

For pair-wise comparisons Welch's t-tests and/or Wilcoxon's rank sum tests were performed (after a repeated measures ANOVA). For classification, random forest analyses were done. Statistical analyses were performed using the program “R”.

#### Selection of Analytes for Focused Statistical Analysis:

We conducted a PubMed search (2020/04/01) using the phrase “(mouse OR rat) AND (germ-free OR antibiotic) AND [(serum OR plasma OR blood)] AND metabolome.” We evaluated the resulting articles as well as any additional relevant publications that they referenced. Antibiotic reports were considered to be relevant if they met the following criteria, i) they used a conventional animal strain, ii) the antibiotic was administered for > 6 days, iii) a control group not treated with antibiotic was included, iv) animals were maintained on a standard lab diet, and v) blood was drawn no later than 24 hours after completion of antibiotic treatment. Our final focused list consisted of chemicals whose serum levels were reported to be affected both by GF status and by treatment with at least 1 antibiotic.

From the data provided by Metabolon©, we decided *a priori* to exclude those chemicals for which > 50% of baseline data were missing. Given that antibiotics often suppress GMB-derived metabolites, chemicals with > 50% missing values at the post-antibiotic time point were included. To address fluctuations that can occur for reasons other than antibiotic treatment, we also decided *a priori* to exclude those chemicals which showed significant changes between any of the time points, PRE-, MID-, and POST-antibiotic in the control group. Finally, chemicals that were significantly different between the control group and antibiotic group at the PRE time point were also to be excluded *a priori*.

The list of chemicals obtained from the PubMed search was cross-referenced with the list of chemicals we measured in patient serum subject to the *a priori* exclusions. Chemicals common to both lists were subjected to planned t-test comparisons with significance set at *P* < 0.05, uncorrected for multiple comparisons.

## RESULTS

A total of 20 antibiotic-treated patients (mean age 70.5 years, range 50-90 years, all male) and 5 controls (mean age 75.4 years, range 64-90 years, all male) completed the study. With the exception of 1 antibiotic-treated participant who provided 2 MID blood samples and no POST sample, all other participants provided PRE, MID, and POST samples. The antibiotic treatment regimens included a broad-spectrum beta-lactam antibiotic (18 piperacillin/tazobactam, 1 ertapenem, and 1 ceftriaxone) alone in 4 patients, and in combination with additional agents in 16 patients (14 intravenous vancomycin and 2 moxifloxacin). The median duration of antibiotic treatment was 5 days (range 2-20 days).

### General Analysis

The metabolomic analysis provided putative identification for 499 chemicals. Under general analysis, 69 chemicals in the control group and 153 chemicals in the antibiotic group showed significant uncorrected POST vs PRE differences (Supplementary Table 1), but in no case did any reach the FDR required for a significant time x treatment interaction (Supplementary Table 1, Supplementary Data). The Hierarchical Clustering and a Principal Component Analysis showed a high overlap between the groups (Supplementary Figure 2). The Random Forest Analysis showed that the control group and the antibiotic-treated groups could be separated before, during, or at the end of the study with a predictive accuracy 65%-68% (Supplementary Figure 3). However, none of the chemicals identified by the biochemical importance plot (Supplementary Figure 4) could sustain the FDR for a significant time x treatment interaction (Supplementary Data [Pathway Heat Map]).

### Focused Analysis

Our PubMed search identified 8 relevant studies of antibiotic-effects on serum chemical levels in mice or rats [43–50] (Table 1). We also found 7 mouse studies [1, 51–56] that cumulatively identified 21 unique chemicals affected by GF status (Table 2). Overall, we identified 10 serum chemicals reported to be affected both by GF status and by antibiotic treatment in rodents.

**Table 1:** Antibiotic Effects on Small Molecules in the Serum of the Mouse or Rat

Source	model	Antibiotic	Duration (Days)	Day(s) of blood collection
[43]	rat, M	neomycin or gentamicin or moxifloxacin or levofloxacin or doxycycline or tetracycline	28	7, 14, 28
[44]	rat, M/F	clindamycin or lincomycin	28	7, 14, 28
[45]	rat, F	mequindox (low/med/high dose)	91	35, 91
[46]	rat, M	vancomycin or (streptomycin + bacitracin + polymyxin B)	7	0, 7
[47]	mouse, M	ampicillin + vancomycin + neomycin + metronidazole	28	28
[48]	rat, M	penicillin	14	0.5, 1, 14
[50]	mouse, M	ampicillin + vancomycin + neomycin + metronidazole	14	15
[49]	mouse, M	ampicillin + neomycin	28	28

Mouse or rat studies in which serum chemicals were reported and which satisfied the following criteria: i) conventional strain, ii) antibiotic administered for > 6 days, iii) included a non-antibiotic treated control group, iv) standard lab diet, v) blood drawn no later than 24h after the termination of antibiotic. M- male, F- female.

**Table 2:** Selecting Small Molecules for Focused Analysis in Serum Based on their Sensitivity to Germ-Free Status and Antibiotic Effects in Rodents

Serum Levels of	Effect of Germ-Free status (mouse)	Effect of Abx (mouse or rat)	Measured in current study	Included in Focused Analysis
cinnamoylglycine	✓-		✓	
diydroxyquinoline glucuronide	✓-			
equol sulfate	✓-			
hippuric acid*	✓	✓	✓	✓
indole-3-propionic acid*	✓-	✓	✓	✓
indoxyl-3-sulfate*	✓-	✓	✓	✓
methyl equol sulfate	✓-			
N-acetyltryptophan	✓	✓	✓	α
p-cresol sulfate*	✓-	✓	✓	✓
phenol sulfate*	✓-	✓	✓	✓
phenylacetylglycine	✓	✓		
phenylpropionylglycine	✓-			
pyrocatechol sulfate	✓	✓		
serotonin*	✓	✓	✓	✓
L-tryptophan	✓		✓	
L-tyrosine	✓	✓	✓	β
urate	✓		✓	
3-(3-sulfooxyphenyl) Phenyl sulfate propanoic acid	✓-			
3-Carboxy-4-methyl-5-pentyl-2-furanpropionic acid glucuronide	✓			
5-hydroxyindoleacetic acid	✓			
12-Hydroxy-5Z,8Z,10E,14Z,17Z-eicosapentaenoic acid	✓			

Serum chemicals affected by germ-free status [1, 51–56], were cross-referenced with those affected by antibiotics in mouse or rat studies [43–50] and with those for which data from the current human study were available. – serum levels not detectable in GF animals, α - > 50% data missing at a relevant time point, β - trend towards significant difference between controls and antibiotic-treated group at the PRE time point

* accepted for focused analysis.

**Figure 1.**
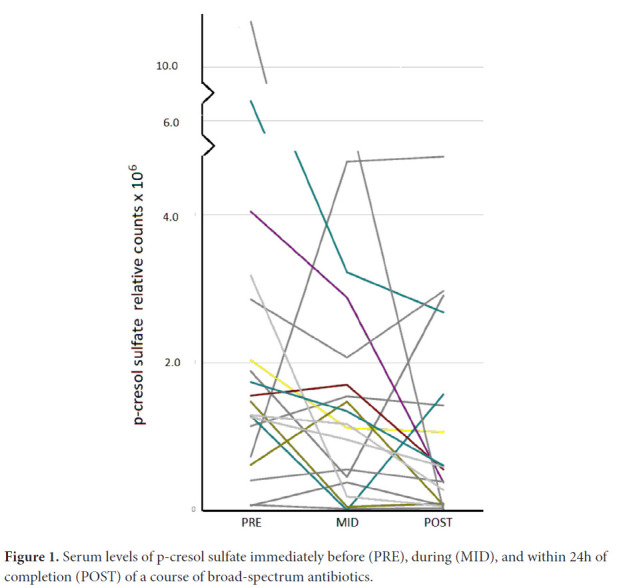
Serum levels of p-cresol sulfate immediately before (PRE), during (MID), and within 24h of completion (POST) of a course of broad-spectrum antibiotics.

Eight such chemicals were among those measured in our human serum (Table 2, Supplementary Data [Pathway Heat Map]). N-acetyltryptophan was then excluded because > 50% of the data were missing at 1 time point. L-tyrosine was excluded because its levels at the PRE-time point tended to be higher in the antibiotic treatment group. Thus, 6 chemicals remained for consideration (Table 2). Of those, only levels of serotonin (5HT) did not show significant differences in POST vs PRE or MID vs PRE comparisons whereas levels of 5 chemicals significantly declined (Table 3). The pattern of decline over time, however, varied greatly across individuals. In the case of p-cresol sulfate, for instance, some individuals showed progressive decline over the duration of treatment, others showed maximal decline at the MID point with some recovery, and several showed a decline only after the MID point (Figure 1).

**Table 3:** Effects on Serum Levels of Small Molecules: Antibiotics in Patients vs Germ-Free Status or Antibiotics in Animal Models

chemical	Serum Ratios				*P-values*		
	Human Abx MID/PRE, POST/PRE	Mouse GF/CONT	Rat or Mouse Abx/CONT	Rat or Mouse Abx regimen	Human Abx	Mouse GF	Rat or Mouse Abx
p-cresol sulfate	**0.49**, 0.66	nd^b^,^g^	0.09^e^	VAN	0.002	0.002	< 0.001
			0.11^e^	STREP + NEO + BACI + POLY			< 0.001
			.04^l^	AMP + NEO + METRO + VAN			< 0.001
phenol sulfate	**0.34, 0.62**	nd^b^	0.04^e^	VANCO	0.003	10^−6^	< 0.001
		0.05^a^-^g^	0.03^e^	STREP + NEO + BACI + POLY		0.002	< 0.001
hippurate	0.76, **0.46**	0.06^b^	0.10-0.30^c^	LEVO	0.05	2 × 10^−9^	< 0.2*
			0.17-0.43^c^	MOXI			< 0.2*
			.09-0.15^c^	DOX			< 0.2*
			0.16-0.35^c^	TETRA			< 0.2*
			0.20-0.45^c^	NEO			< 0.2*
			0.12-0.54^c^	GENT			< 0.2*
			0.05	PEN			< 0.05
			0.06^e^	VAN			< 0.001
			0.06^e^	STREP + NEO + BACI + POLY			< 0.001
			.07-.13^d^	CLIN			< 0.2*
			.033-1.97^d^	LINCO			< 0.2*
indole propionate	1.18, **0.45**	nd^b^	0.12-0.30^c^	NEO	0.005	8 × 10^−7^	< 0.2*
			.06-0.42^c^	GENT			< 0.2*
			0.37^e^	VANCO			< 0.001
			0.44^e^	STREP + NEO + BACI + POLY			< 0.001
			.01^l^	AMP + NEO + METRO + VAN			< 0.001
indoxyl sulfate	**0.57, 0.54**	nd^a^^,^^b^^,^^g^^,^^h^^,^^i^	0.25-0.68^c^	DOX	0.02	1.3 } 10^−7^^b^, 0.002^a^	< 0.2*
			0.33-0.51^c^	TETRA			< 0.2*
			.01-.025^f^	PEN			< 0.05
			0.47-0.66^e^	STREP + NEO + BACI + POLY			< 0.001
			.44^l^	AMP + NEO + METRO + VAN			< 0.001
serotonin	0.97, 0.95	0.33-0.36^b^^,^^j^	3.11^e^	VAN	0.14	1.3 × 10^−10^^b^^,^^j^	< 0.001
			0.52^k^	AMP + NEO			

Serum ratios (POST/PRE or MID/PRE) of antibiotic (Abx)-treated patients or germ-free (GF) or Abx-treated animals/controls (CONT). BACI – bacitracin, CLIN – clindamycin, DOX – doxycycline, GENT – gentamicin, LEVO – levofloxacin, LINCO – lincomycin, MOXI – moxifloxacin, NEO – neomycin, PEN – penicillin G, POLY – polymyxin B, STREP- streptomycin, TETRA – tetracycline, VAN- vancomycin.

a Chu *et al*, 2019

b Wikoff *et al* 2009

c Behr *et al*, 2017

d Behr *et al* 2018

e Lam *et al*, 2016

f Sun *et al*, 2013

g Devlin *et al*, 2016

h Mishima *et al*, 2017

i Shimada *et al*, 2013

j Sjogren *et al*, 2012

k *et al*, 2019

l Cho Y *et al*, 2019)

nd - not detected in GF animals. P-values were calculated for means. POST vs PRE or MID v PRE for human Abx, GF v CONT, Abx v CONT for animal studies. Bold values for Human Abx designate comparisons which were statistically significant p < .05.

* Welch's t-test at p < 0.2 considered significant.

## DISCUSSION

In our group of patients treated with broad-spectrum antibiotics, serum levels of 5 of 6 chemicals predicted to be highly GMB-dependent by animal studies, declined significantly as measured by univariate analysis. While consistent with our hypothesis, the findings have to be critically interpreted in terms of assumptions, experimental limitations, and generalizability.

We enlisted inpatient male veterans with any of several medical conditions for which broad-spectrum antibiotic treatment was indicated. The duration of antibiotic treatment varied considerably. Our small sample of controls was not clinically matched with the treatment group. We used a commercial metabolomic service (Metabolon©) that conducted a non-targeted analysis using GC/MS or LC/MS/MS. While such an approach supports detection of a very large number of chemicals, it does so on the basis of available spectroscopic libraries rather than by direct comparisons to authentic standards. This yields presumptive identification and semi-quantitative comparisons [39]. On the other hand, the chemicals in our focused analysis have relatively simple structures and well-characterized spectral properties.

A classical ANOVA that included all the aggregate data (20 patients, 5 controls, 3 time points, 499 chemicals) did not show any time x treatment interactions that exceeded the preset q < 0.1. Part of this was due to the FDR for multiple corrections. However, even if the q value was ignored and any time x treatment interaction *P* < 0.05 accepted, relatively few analytes would have reached significance. This can be partially attributed to the high variance of the data as well as to the unequal sizes of the control (n = 5) and antibiotic-treated (n = 20) groups. We dealt with these issues by imposing criteria determined *a priori* to restrict the number of chemicals admitted to a focused univariate analysis.

The requirement that serum levels of a given chemical show GMB-dependence in both GF and antibiotic animal studies had by far the largest overall impact. Our PubMed search identified only 10 such serum chemicals (Table 2). Of those, phenylacetylglycine and pyrocatechol sulfate were not part of the panel of chemicals measured in our human serum (Table 2). Thus, only 8, or 1.6% of the total number (n = 499) of putatively identified chemicals could be considered for a focused evaluation. We also decided *a priori* to exclude chemicals whose levels in the control group showed significant changes during the experimental period. It turned out, however, that this rule had no practical effect and did not further limit the chemicals identified as antibiotic-responsive in animal studies (Table 2). Second, we decided to exclude chemicals that at the PRE (ie, baseline) time point already tended to be significantly different (*P* < 0.1) between controls and antibiotic-treated individuals. Such differences may be attributable to chance, underlying illness, or other factors. L-tyrosine was excluded in this way. Third, we decided to exclude all chemicals for which > 50% of data were missing for any time point in the controls or at the PRE time point in the antibiotic group. This was based on the fact that GF-status [1, 52, 53] or antibiotic treatment [43–48] was much more likely to lower serum levels of a chemical than to elevate it (Table 3). Hence, failure to detect a chemical after initiation of antibiotic treatment could reflect antibiotic-induced suppression of that chemical. However, had the > 50% missing data exclusion been applied universally, it would not have affected the exclusion of N-acetyltryptophan, for which > 80% of levels were unavailable across all time points in both groups (Supplementary Data).

### Detection of GMB Effects on the Metabolome – Comparative Approaches

Of the roughly 10^14^ cells comprising the human organism ~ 90% are exogenous, with the vast majority contributed by the GMB [57]. Cell density in the colon exceeds that for any other known ecosystem [58]. To meet its bioenergetic needs, each gut microbe must generate energy, maintain a redox balance, and acquire carbon and nitrogen for synthesis of necessary chemicals [59]; to achieve this, it must import needed substrates and export unwanted products. Intraluminally generated chemicals which cross the gut wall become constituents of the host's metabolome. Accordingly, attempts to define the GMB-sensitive metabolome often start with identification of chemicals that individual microbes can absorb, metabolize, synthesize and then release into the gut lumen.

Taxonomic profiling, commonly based on deep sequencing of 16s rRNA, identifies the type and number of elements of the GMB. Functional profiling supports development of integrated catalogues of the human fecal microbial metagenome that characterize the metabolic capacity of the GMB [60]. The latter, in combination with transcriptomics, proteomics, and metabolomics can define which microbial genes are actually expressed under given conditions. Some enzymatic capacities within the human organism are GMB exclusive, others can be located only within endogenous human cells, and the remainder can be expressed in both [61]. A comparison of such GMB-derived data with homologous information about the metabolic potential of endogenous human cells [62] can suggest the origin of given chemicals within the human metabolome. Confirmatory information, however, must be derived from *in vivo* studies.

Homologous data can be readily obtained from gnotobiotic animal models, the extreme being the GF organism, reared from birth without a detectable GMB [7]. Thus, sensitivity to GF status was the initial inclusion criterion for our focused list of chemicals in serum (Table 2, Table 3) [1, 51–56]. Of the 21 chemicals so identified, 10 were not detectable or almost undetectable in the GF state (Table 2) and hence likely to be exclusively dependent on the GMB.

The presence of a healthy GMB is critical to the development of multiple systems including internal barriers (gut-blood, blood-brain) and end organs; the GF rodent is not just a normal animal in which the GMB has suddenly been eliminated [7]. Furthermore, the GF state does not model the multiple adaptive changes that take place within a GMB exposed to antibiotics. Such primary antibiotic-induced effects on GMB architecture can secondarily affect luminal levels of chemicals absorbed or secreted by the antibiotic-adapted GMB [10, 11, 22]. For instance, when antibiotic treatment suppresses certain constituents of the GMB, hypervirulent strains of *C. difficile* can exploit that niche by generating concentrations of p-cresol which are bacteriostatic to other microbes [63–65]. Alternatively, one group of microbes can provide chemicals needed by a second group of microbes which in turn promotes an environment favorable to the growth of the first [66]. Furthermore, the GMB is characterized by a high degree of metabolic redundancy; most metabolic pathways are encoded within functional genetic categories distributed across multiple types of bacteria rather than being exclusive to one genus or strain [61, 67]. Thus, antibiotics are unlikely to suppress or eliminate metabolites to the extent achieved by GF status.

For these reasons, we imposed an additional constraint on our focused list of samples, namely that serum chemicals had to be affected by antibiotic treatment in rodents. Although GMB diversity in the mouse is relatively closer to that of man [68, 69] we accepted both mouse and rat studies. We also accepted studies using any combinations of male or female animals, sexual dimorphism notwithstanding [44, 53, 70]. We did not consider metabolome data derived from matrices other than serum, given that changes across these compartments (eg, urine, feces) often correlate poorly [44, 45, 71].

### Human Studies of Antibiotic Effects

A PubMed Search (initial search terms: [(serum OR plasma OR blood) AND antibiotic AND metabolome AND human]) yielded only 5 heterogeneous human serum studies of antibiotic effects [14–17]. An 8-week treatment of patients (n = 20) with cirrhosis and minimal hepatic encephalopathy with rifaximin, a poorly absorbed antibiotic with minimal effect on the GMB [18], elevated serum levels of several saturated as well as unsaturated fatty acids [72]. In the only available double-blind study, overweight or obese men (n = 57) were randomly assigned to receive placebo, amoxicillin (broad-spectrum), or vancomycin (narrow-spectrum) for 7 days; a very small number of serum chemicals were measured [14]. Vancomycin treatment elevated fecal primary bile acid levels over 5-fold and lowered fecal butyrate to below 20% of pre-treatment levels, but those changes were not reflected in serum [14]. In an open study, patients with chronic obstructive pulmonary disease were randomized to continue standard treatment with a combination of inhaled β-adrenergic agonists and inhaled corticosteroids (n = 60) or to receive add on doxycycline (n = 60) for 90 days [16]. An untargeted metabolomic analysis associated doxycycline treatment with elevations of serum citrate, imidazole, and L-arginine but a lowering of lactate and an unspecified fatty acid [16]. Of note, levels of several serum metabolites also rose in the control group [16]. In a recent study, blood samples drawn early (0 – 2 days) and late (25 days +) during treatment of infective endocarditis with any of several antibiotics, showed a lowering of several amino acids (L-tyrosine, L-valine, L-leucine, L-isoleucine) and other chemicals (glucose, mannose, unspecified polyol) [17]. Overall, the heterogeneity of antibiotics, duration of treatment, and panels of evaluated chemicals in these studies does not justify generalized conclusions.

### Interpreting Antibiotic-Associated Serum Metabolome Changes

**Figure 2.**
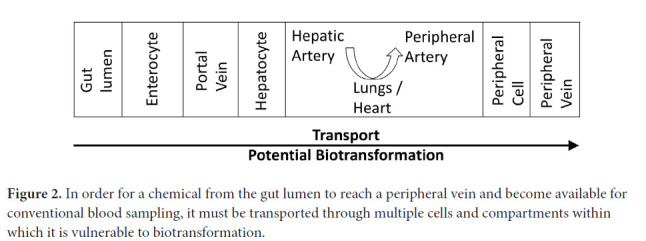
In order for a chemical from the gut lumen to reach a peripheral vein and become available for conventional blood sampling, it must be transported through multiple cells and compartments within which it is vulnerable to biotransformation.

There are many steps between the appearance of a molecule in the gut lumen, whether as an endogenous or microbial product, and its entry into peripheral serum (Figure 2). Transport across cell walls and other barriers may be passive or active and affected by a number of genetic and other factors [34]. Biotransformations may take place in the intestinal lumen, in the enterocytes lining the lumen, in the liver, kidney, or other organs [34]. Hence, both transport and biotrans-formation of chemicals must be considered when interpreting changes in the antibiotic-affected serum metabolome. To minimize possible primary endogenous metabolic factors, we excluded patients with end-stage renal or hepatic disease. While, in general, antibiotics have larger effects on the GMB than on hepatic or renal function or on transport mechanisms [73–75], each chemical of interest should be evaluated individually.

### Phenolic Metabolites

The serum chemicals we identified as antibiotic-sensitive in man belong to the phenolic (hippu-rate, p-cresol sulfate, phenol-sulfate) or indolic (indoxyl sulfate, indole propionate) pathways. Hippuric acid is formed through the conjugation of benzoic acid (BA) with the hydrophilic moiety glycine [76, 77] in a phase II process facilitating removal of the hydrophobic but potentially toxic BA [78, 79]. Benzoic acid itself cannot be synthesized by endogenous human enzymes [62]. On the other hand mixed fecal cultures or certain constituents of the human GMB can, under anaerobic conditions, generate BA or its hydroxylated metabolites from a variety of precursors, including free L-tyrosine or L-phenylalanine [80, 81], 4-hydroxyphenylpyruvic acid [81], polyphenol rich dietary components (eg, berries, black tea, bran, fruit juices, wine) [82–89], or pure polyphenols themselves (eg, catechin, epicatechin, proanthocyanidins, quercetin) [90–93].

While HA and its hydroxylated metabolites are constituents of the human fecal metabolome [94], the GMB cannot glycinate BA to generate HA. In man, such glycination has been demonstrated in the liver and kidney [77, 95], but whether it can also take place within enterocytes, as is the case in the rat [96], is not known. In any case, the conjugation occurs via a coenzyme-A-dependent process localized to the mitochondrial matrix [97].

Similarly, neither phenol nor p-cresol can be synthesized in appreciable quantities by endogenous human enzymes [62] but can be generated under anaerobic conditions by GMB from free L-tyro-sine [80, 81, 98–102] or dietary polyphenols [103]. Phenol and p-cresol are considered end-products of the GMB in that they are not further broken down in the intestinal lumen [104]. Both occur in human serum almost exclusively as sulfated conjugates (Supplementary Data) with p-cresol glucuronide a minor metabolite [105]. Aryl sulfotransferase activity capable of sulfating phenol is expressed in human feces [106] and can be suppressed by antibiotics in animal models [107]. In man, phenol-sulfotransferase activity is expressed within tissue of the small intestine [108, 109], even more so in liver, and more modestly in the kidney [109, 110]. Colonic tissue remains to be similarly evaluated. Thus, while both phenol and p-cresol are generated exclusively by the GMB, the relative importance of multiple potential loci of sulfation is not known. Antibiotic effects on their sulfation or glucuronidation are not known.

While antibiotic treatment is not known to affect conjugation of BA or p-cresol; effects on their transport must be considered separately. Gut luminal BA crosses the basilar membrane of enterocytes via monocarboxylic transporters [111, 112]. Antibiotics can markedly suppress GMB-mediated synthesis of short chain fatty acids [113], which in turn, can act both as substrates and inducers for colonocyte monocarboxylic transporters [114]; thereby potentially affecting transport of other substrates such as BA. The transport of free p-cresol across the human gut wall remains to be similarly characterized, but in general, fecal levels of free p-cresol correlate with serum levels of its conjugates [115].

Sulfated conjugates of phenol and p-cresol can be detected in human feces [115, 116]. In addition, p-cresol sulfate can be taken up by Breast Cancer Resistance Protein members of the ATP-binding cassette (ABC) protein transporter family [117] expressed on the apical membrane of human colonocytes [118, 119], as well as by organic anion transporters (OAT) (hOAT1, hOAT3) on human kidney cells [120–124]. The expression of hOAT3 in tissue of the small intestine has been detected by some investigators, but not by others [119, 125, 126], and hOAT1 and hOAT3 may also be expressed in rectal tissue [127]. Certain antibiotic (eg, penicillins, cephalosporins) can inhibit OAT-mediated uptake of substrates [128–131]. However, this would limit renal excretion of such chemicals and; hence, elevate their serum levels. Since levels of our targeted analytes were lowered by antibiotic treatment, antibiotic effects on OAT-mediated transport are unlikely.

### Indolic Metabolites

Constituents of the human GMB readily convert L-TRP to indole [80, 104, 132–134]; endogenous human enzymes cannot do so [62]. Indole is the most abundant L-TRP metabolite of the normal human fecal metabolome (~ 3mM) [3, 135] and its levels correlate with those of serum indoxyl sulfate [115]. The conventional wisdom is that intraluminally-formed indole is transported across the colonic wall, enters the portal circulation and reaches hepatocytes, where microsomal cytochrome CYP2E1 3-hydroxylates the indole to form indoxyl [136, 137]. The latter, in turn can be sulfated to indoxyl sulfate, secreted into blood [138] and then cleared by the kidney. However, indoxyl sulfate itself is a constituent of the human fecal metabolome [115], raising the possibility that some fraction of it is either generated within the GMB or is secreted into the lumen by the host. Tracer studies in the rat, for instance, show that a small fraction of systemically administered indole enters the bile as indoxyl sulfate [139]. Furthermore, indoxyl sulfate taken up from the systemic circulation can be directly secreted into the intestinal lumen under conditions of renal failure [140]. Intriguingly, a recent rat study found no significant differences in postprandial levels of indoxyl sulfate between the portal and systemic circulations [141]. This suggests that under usual conditions, most plasma indoxyl sulfate is generated within the GMB and/or enterocytes, without a significant hepatic contribution. Comparable studies in primates have not been conducted. In any case, indoxyl sulfate is cleared mainly through renal excretion mediated at least in part by hOAT3, in a process that can be inhibited by ciprofloxacin [131]. While 2 of our patients were treated with moxifloxacin, another quinolone antibiotic, any inhibition would elevate, not lower indoxyl sulfate levels as we observed in our data.

The kinetics of indole propionic acid are less well characterized. While human cells can successively metabolize L-TRP to indole-3-pyruvic acid and then to indole-3-lactic acid [62, 142, 143], only certain constituents of the GMB are known to metabolize L-TRP through to indole propionic acid [133, 143–147]. That reductive pathway may involve indole-3-lactic acid, indol-3-acrylic acid, or other intermediates [143, 148]. Indole propionic acid can inhibit the human proton-coupled amino acid transporter in the intestinal epithelium but is not itself a transport substrate for it [149]. Thus, the transport of indole propionic acid across the gut wall remains to be characterized. In man, indole propionic acid does not undergo phase II metabolism and has been detected only as the parent compound [3]. Antibiotic effects on the transport of indole propionic acid are not known.

5HT was the only chemical whose serum levels were affected both by GF and antibiotic status in rodents (Table 2) but which was not significantly affected by antibiotic treatment in our focused analysis (Table 3). It is also the only chemical in our list that can be generated endogenously by mammalian cells [62]. Isolated reports that certain GMB constituents can synthesize 5HT directly from L-TRP [150] notwithstanding, there is no evidence that GMB-derived 5HT significantly contributes to serum levels of 5HT. On the other hand, over 90% of endogenous 5HT in the rat is synthesized by various components of the gut, namely, mucosal mast cells and myenteric neurons and, particularly, enterochromaffin cells, which express tryptophan hydroxylase 1, the rate-limiting enzyme for 5HT synthesis [151]. The gut-generated 5HT is taken up by platelets and distributed systemically [151]. Estimates from rodent models suggest that while over 80% of serum 5HT originates in cells endogenous to the gut of the host [152], about 50% of serum 5HT is subject to some regulation by the GMB [153].

Short chain fatty acids (SCFAs), generated via GMB-mediated fermentation of dietary fiber and protein, upregulate tryptophan hydroxylase 1 [154, 155]. Since SCFAs are absent from the intestinal lumen of the GF mouse [156, 157], GF status is associated with lower expression of tryptophan hydroxylase 1 in colonic tissue [56, 154]. This, given the outsized contribution of enterochromaffin cells to serum 5HT [151], is consistent with lower serum levels of 5HT in the GF mouse [1, 56]. Antibiotic effects in animal models are more difficult to explain. Treatment with vancomycin, an antibiotic that relatively selectively suppresses enterococci [158] produced a 3-fold elevation of serum 5HT in the rat [46]. However, changes in serum 5HT levels did not reach significance after treatment with combinations of antibiotics ([streptomycin + neomycin + bacitracin + polymyxin [46])], [ampicillin + neomycin + metronidazole + vancomycin [50] ]) intended to sterilize the gut. In contrast, a combination of ampicillin and neomycin lowered serum 5HT levels in the mouse by almost 50% [49]. The precise mechanisms that would explain these differences remain to be determined.

### Comparison to Effects of Ileostomy

In developing our *a priori* criteria for selecting chemicals of interest we did not include data from patients with ileostomies. While the overwhelming preponderance of the total GMB reside in the colon, the bacterial density in the terminal ileum actually exceeds that of the large intestine [159]. Furthermore, a sparse microbiota can be found throughout the normal digestive tract [160, 161]. Naso-ileal tube aspirates from patients with intact colons reflect a similar GMB composition to that in ileal fluid from patients with ileostomies [162]. The enzymatic activity within ileal fluid is capable of metabolizing the flavonoid precursors of multiple phenolic metabolites [163]. At least some members of the ileal GMB express functionally significant tyrosine decarboxylase activity [164]. This suggests that the functional capacity of the remaining GMB in patients with ileostomies could, in theory, contribute to the human serum metabolome. In view of other data, however, that possibility seems remote. In patients with renal failure, colectomy is associated with either negligible (p-cresol sulfate, indoxyl sulfate) or significantly lower (HA) serum levels [165] of 3 of the metabolites identified as unequivocally GMB-dependent in animal studies (Table 2). Comparable data on serum or urine phenol sulfate or indole propionic acid levels after colectomy are not available. Not surprisingly, all the chemicals identified in our focused list are either known or suspected to be uremic solutes [115, 166].

### Associations with Specific Gut Microbiota

Without any direct measures of the GMB, we cannot empirically evaluate possible associations between antibiotic-induced changes in the serum metabolome and concomitant changes in individual operational taxonomic units (OTUs) of the GMB. The available literature supports several generalizations. Most antibiotic-treatment regimens lower the overall diversity of the GMB [14, 167–170]. Furthermore, antibiotic class-specific effects on the GMB architecture have been well demonstrated [8–11]. Our antibiotic regimens included β-lactam agents, which are known to be excreted into the intestinal tract where they can dramatically alter the GMB [9]. The most common beta-lactam antibiotic treatment was the piperacillin/tazobactam combination, which provides broad-spectrum activity against anaerobes, facultative gram-negative bacilli, and enterococci [9, 10].

Most metabolic pathways are encoded within functional genetic categories distributed across multiple types of bacteria rather than being exclusive to one genus or strain [61, 67, 171]. Suppression of one taxa can create a niche opportunity for other taxa [11, 158, 172]. Furthermore, some enzymatic capacities within the human organism are GMB exclusive, others can be located only within endogenous human cells, and the remainder can be expressed in both [61]. A given GMB-derived chemical in the human metabolome does not *per se* carry the fingerprint of the particular microbe that generated it. Hence, antibiotic-induced lowering of serum chemicals is more likely to be detected for those chemicals generated relatively exclusively by taxa targeted by the antibiotic regimen.

All of the 5 chemicals determined to be antibiotic-responsive in our study are themselves either fermentation products or metabolites of fermentation products of aromatic amino acids. Although aromatic amino acids can be metabolized to some degree by cultures of a range of intestinal anaerobes, including members of the genera *Bacteroides, Lactobacillus, Clostridia,* and *Bifido-bacteria* [173], only limited members of those genera can generate BA, p-cresol, or phenol from L-TYR and/or L-PHE [80, 98–102]. There is further specificity within strains. *Clostridium difficile* can ferment L-TYR, L-PHE, or L-TRP whereas *C. perfringens* can do so only to L-TYR [99, 144]. Furthermore, *C. difficile* is the only *Clostridial* species expressing 4-hydroxyphenylacetate decarboxylase (EC 4.1.1.83), an enzyme critical to the metabolism of L-TYR through to the end product p-cresol [63, 174].

A relatively large number of anaerobic constituents of the healthy human GMB, including *E. coli,* express tryptophanase (EC 4.1.99.1), which converts L-TRP to indole [80, 99, 104, 132–134]. *Clostridium difficile* cannot itself generate indole but can modify the intestinal milieu to promote indole-generation by other bacteria [175]. A more restricted number of microbes, including *Clostridial* strains, can ferment L-TRP to indole propionic acid [99, 133, 143–147].

We noted earlier, that many small phenolic molecules can also be generated by GMB-mediated degradation of polyphenol rich dietary components [82–89]. This applies to all 3 phenolic metabolites in our study (phenol sulfate, p-cresol sulfate, HA). We cannot distinguish the fraction of such GMB-generated metabolites derived from dietary polyphenols vs free L-TYR or L-PHE. The critical first step in polyphenol metabolism is cleavage of the C ring of the heterocyclic flavan nucleus, a reaction outside the capacity of endogenous human enzymes but one that can be carried out by a limited number of members of the human GMB [34], including several *Clostridial* species [176–179]. Regulation of the full cascade of polyphenol metabolism, however, is incompletely understood. For example, while plasma HA levels rise rapidly after the ingestion of black tea polyphenols [180], dietary polyphenols either lower or do not affect the excretion of phenol sulfate [181, 182]. A switch from a conventional Western diet to an uncooked but fiber- and polyphenol-rich vegan diet with comparable total protein content lowers both serum phenol sulfate and p-cresol sulfate [183]. This underscores that dietary constituents other than polyphenols can also affect serum levels of phenolic end products. Whether the same members of the GMB mediate all such processes remains to be determined.

Of course, data from *in vitro* monoculture experiments must be complemented by *in vivo* studies that allow for polymicrobial population dynamics. Such efforts remain highly preliminary. In a subsample (n = 855) of individuals with minimal renal decline, serum indoxyl sulfate and p-cresol sulfate were both associated with the presence of family members of *Clostridiales* in the GMB [184]. More recently, in a study of mixed inpatients treated clinically with antibiotics, uri-nary indoxyl levels were found to be associated with the relative abundance of *Clostridiales* [168]. Thus, our own data would suggest that the broad-spectrum antibiotic suppressed at the very least, members of the *Clostridia* family.

## CONCLUSIONS

Using a clinical sample of patients and a limited number of targets we demonstrated that broad-spectrum antibiotic treatment lowered serum levels of 5 chemicals (phenol sulfate, p-cresol sulfate, HA, indoxyl sulfate, indole propionic acid) previously shown to be absent or markedly suppressed in the serum of GF mice (Table 3). Based on a consideration of transport and other mechanisms, we conclude that the most likely explanation for the data is an antibiotic-induced suppression of *Clostridia* and other species. All 5 chemicals, often referred to as uremic solutes were previously considered to be GMB-dependent based on various animal and human studies [115, 166]. However, ours is the first study to demonstrate that clinical administration of broad-spectrum antibiotic regimens, even for a variable period of time, significantly lowers serum levels of this group of chemicals. That is all the more remarkable given the manifold other factors known to affect GMB composition and which were not controlled in our study; these include age [26], socioeconomic status [27], genetics [28], concomitant medications [32], and particularly diet [29] and colonic transit time [31]. It is of note that in control populations, excretion of p-cresol sulfate is positively associated while phenol sulfate is negatively associated with colonic transit time [31]. The fact that serum levels of both these chemicals were lowered in our study would indicate that the effects of antibiotic-induced suppression of the GMB were larger than those of other factors which we did not control for.

In view of the increasing use of antibiotics worldwide [19], metabolomic and microbiomic investigations of such treatments remain grossly underutilized. Establishing core chemicals or profiles of such chemicals that confirm antibiotic effects should permit identification of other small molecules that respond more subtly to antibiotic-induced shifts in the GMB. Development of restricted lists of chemicals to be examined *a priori* could allow detection of effects that would not be apparent if statistical adjustments for comparing a very large number of variables were applied. Rapidly emerging data implicating the microbiome and its dependent metabolome across medical disciplines, including immunology [185] and neuropsychiatry [186], oblige investigators to judiciously exploit available patient samples in the service of developing novel biomarkers and treatments.

This work was supported by the Research Service of the VA Northeast Ohio Healthcare System and by a grant from the VISN 10 Research Initiative Program (to CJD and GEJ). The manuscript content is solely the responsibility of the authors and does not necessarily represent the official views of the Department of Veterans Affairs and/or the authors' employers and/or their academically affiliated institutions.

## SUPPLEMENTARY DATA

Supplementary materials are available at the *Pathogens and Immunity* website. Supplementary data may be provided by the authors to benefit the reader. Supplementary data are not copyedited and are the sole responsibility of the authors. Questions or comments related to supplementary materials should be addressed to the corresponding author.
