# Supplementary material for: Changes in the Serum Metabolome of Patients Treated With Broad-Spectrum Antibiotics
Source: Pathog Immun. 2020 Dec 29;5(1):382–418. doi: 10.20411/pai.v5i1.394 (PMC7810407; doi:10.20411/pai.v5i1.394)
Supplement: Supplementary Information, Methods [file pai-5-382-s01.pdf]

Supplementary Information

Methods

Sample Preparation and Analysis

Samples were extracted and prepared for analysis using a standard solvent extraction method. The extracted samples were split into equal parts for analysis on the GC/MS and LC/MS/MS platforms. Also included were several technical replicate samples created from a homogeneous pool containing a small amount of all study samples. Instrument variability was determined by calculating the median relative standard deviation (RSD) for the internal standards that were added to each sample prior to injection into the mass spectrometers. Overall process variability was determined by calculating the median RSD for all endogenous metabolites (ie, non-instrument standards) present in 100% of the Client Matrix samples, which are technical replicates of pooled client samples.

Samples were prepared using the automated MicroLab STAR® system (Hamilton Company, NV). Recovery standards were added prior to the first step in the extraction process for quality control. Sample preparation was conducted using a proprietary series of organic and aqueous extractions to remove the protein fraction while allowing maximum recovery of small molecules. The resulting extract was divided into 2 fractions; 1 for analysis by LC and 1 for analysis by GC. Samples were placed briefly on a TurboVap® (Zymark, MA) to remove the organic solvent. Each sample was then frozen and dried under vacuum. Samples were then prepared for the appropriate instrument, either LC/MS or GC/MS.

For quality control purposes, a number of additional samples were included with each daily analysis. Furthermore, a selection of quality control compounds was added to every sample, including those under test. These compounds were selected so as not to interfere with the measurement of the endogenous compounds. These quality control samples were primarily used to evaluate the process control for each study as well as aiding in the data curation.

Accurate Mass Determination and MS/MS fragmentation (LC/MS), (LC/MS/MS): The LC/MS portion of the platform was based on a Waters ACQUITY UPLC and a Thermo-Finnigan LTQ-FT mass spectrometer, which had a linear ion-trap (LIT) front end and a Fourier transform ion cyclotron resonance (FT-ICR) mass spectrometer back end. For ions with counts greater than 2 million, an accurate mass measurement could be performed. Accurate mass measurements could be made on the parent ion as well as fragments. The typical mass error was less than 5 ppm. Ions with less than 2 million counts require a greater amount of effort to characterize. Fragmentation spectra (MS/MS) were typically generated in a data-dependent manner, but if necessary, targeted MS/MS could be employed, such as in the case of lower level signals.

Bioinformatics

The informatics system consisted of 4 major components, the Laboratory Information Management System (LIMS), the data extraction and peak-identification software, data processing tools for QC and compound identification, and a collection of information interpretation and visualization tools for use by data analysts. The hardware and software foundations for these informatics components were the LAN backbone and a database server running Oracle 10.2.0.1 Enterprise Edition.

LIMS: The purpose of the Metabolon© LIMS system was to enable fully auditable laboratory automation through a secure, easy to use, and highly specialized system. The scope of the Metabolon© LIMS system encompasses sample accessioning, sample preparation and instrumental analysis, and reporting and advanced data analysis. All of the subsequent software systems are grounded in the LIMS data structures. It has been modified to leverage and interface with the in-house information extraction and data visualization systems, as well as third party instrumentation and data analysis software.

Data Extraction and Quality Assurance: The data extraction of the raw mass spectrometer data files yielded information that could be loaded into a relational database and manipulated without resorting to binary large object manipulation. Once in the database the information was examined, and appropriate quality control limits imposed. Peaks were identified using Metabolon© proprietary peak integration software, and component parts were stored in a separate and specifically designed complex data structure.

Compound identification: Compounds were identified by comparison to library entries of purified standards or recurrent unknown entities. Identification of known chemical entities was based on comparison to metabolomic library entries of purified standards. As of this writing, more than 1000 commercially available purified standard compounds had been acquired and registered into LIMS for distribution to both the LC and GC platforms for determination of their analytical characteristics. The combination of chromatographic properties and mass spectra gave an indication of a match to the specific compound or an isobaric entity. Additional entities could be identified by virtue of their recurrent nature (both chromatographic and mass spectral). Proprietary visualization and interpretation software were used to confirm the consistency of peak identification among the various samples. Library matches for each compound were checked for each sample and corrected if necessary.
