# Supplementary figures and images for: Changes in the Serum Metabolome of Patients Treated With Broad-Spectrum Antibiotics
Source: Pathog Immun. 2020 Dec 29;5(1):382–418. doi: 10.20411/pai.v5i1.394 (PMC7810407; doi:10.20411/pai.v5i1.394)

# Changes in the Serum Metabolome

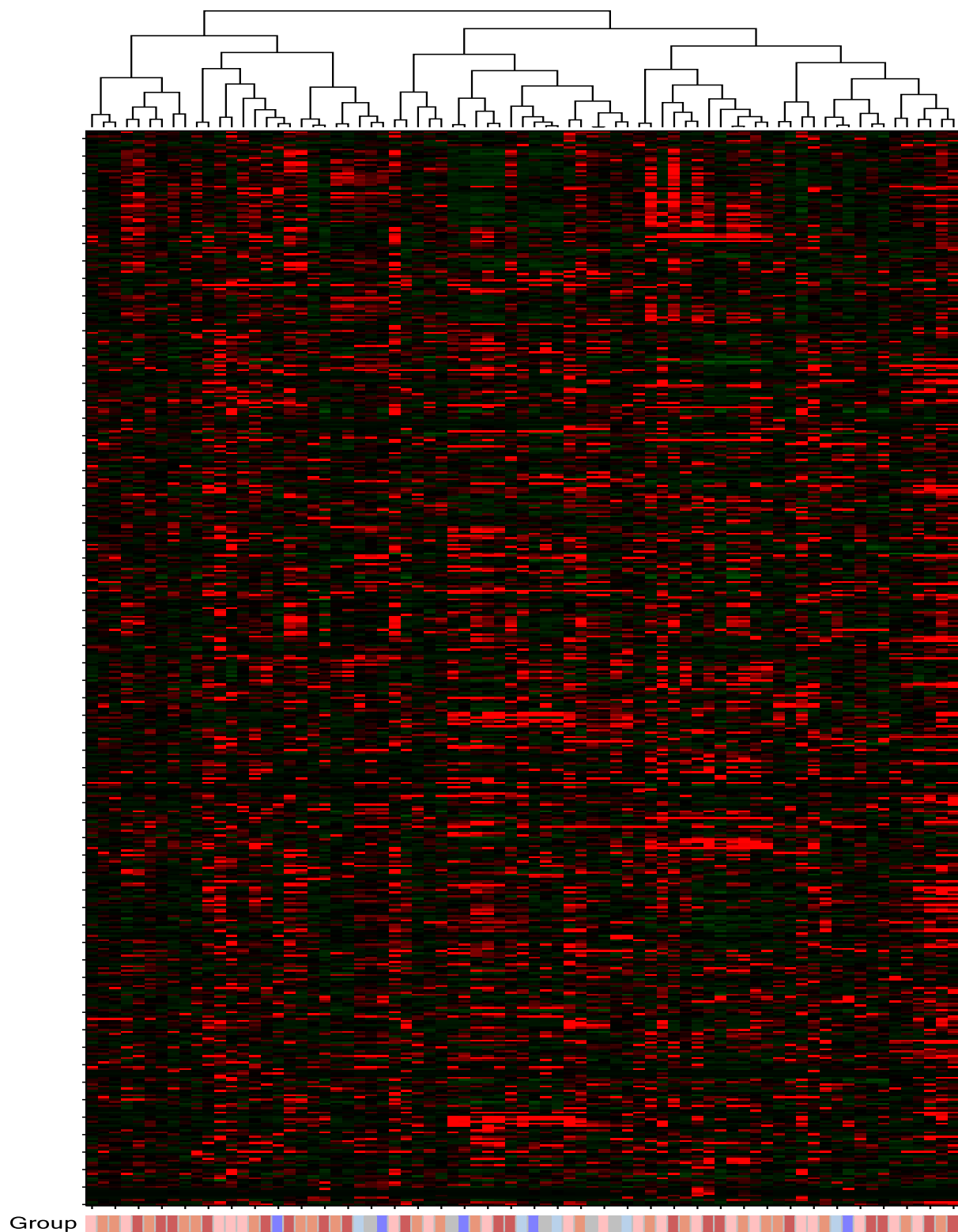

**Figure S1** — Heat Map

Supplement: Supplementary Figure 1 [file pai-5-382-s03.pdf]
