# Supplementary material for: Changes in the Serum Metabolome of Patients Treated With Broad-Spectrum Antibiotics
Source: Pathog Immun. 2020 Dec 29;5(1):382–418. doi: 10.20411/pai.v5i1.394 (PMC7810407; doi:10.20411/pai.v5i1.394)
Supplement: Supplementary Figure 2 [file pai-5-382-s04.pdf]

Changes in the Serum Metabolome

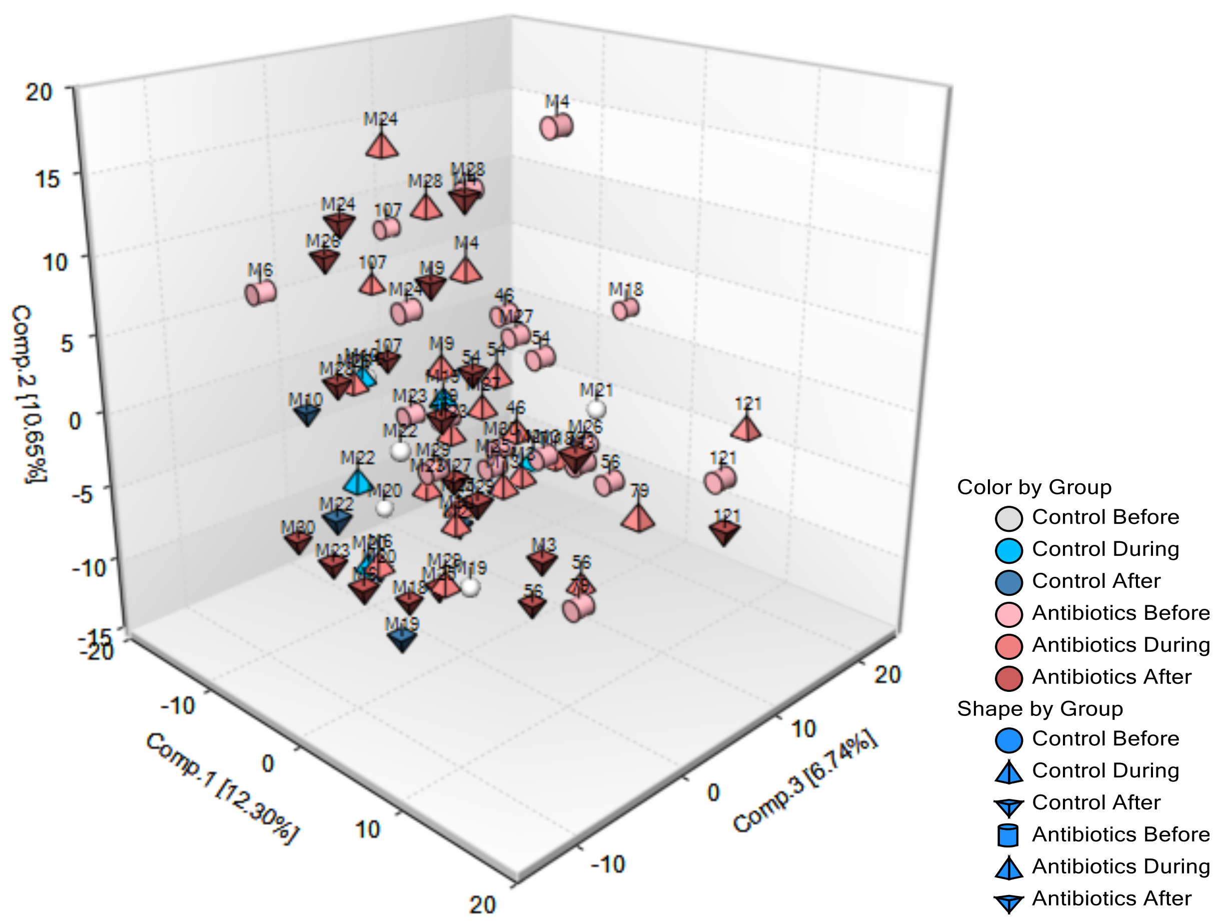

Figure S2 — Hierarchical Clustering and Primary Component Analysis
