# Supplementary material for: Changes in the Serum Metabolome of Patients Treated With Broad-Spectrum Antibiotics
Source: Pathog Immun. 2020 Dec 29;5(1):382–418. doi: 10.20411/pai.v5i1.394 (PMC7810407; doi:10.20411/pai.v5i1.394)
Supplement: Supplementary Figure 3 [file pai-5-382-s05.pdf]

Random Forest Confusion Matrix

Pre-treatment

Predicted Group (columns); Actual Group (rows)

Pre-Tx	Antibiotics Before	Control Before	Class Error
Antibiotics Before	13	7	0.350
Control Before	1	4	0.200
Predictive accuracy = 68%			

Mid-treatment

Predicted Group (columns); Actual Group (rows)

Mid Tx	Antibiotics During	Control During	Class Error
Antibiotics During	13	8	0.381
Control During	1	4	0.200
Predictive accuracy = 65%			

Post treatment

Predicted Group (columns); Actual Group (rows)

Post Tx	Antibiotics After	Control After	Class Error
Antibiotics After	11	8	0.421
Control After	0	5	0.000
Predictive accuracy = 67%			

Figure S3 — Random Forest Analysis
