# Supplementary material for: Changes in the Serum Metabolome of Patients Treated With Broad-Spectrum Antibiotics
Source: Pathog Immun. 2020 Dec 29;5(1):382–418. doi: 10.20411/pai.v5i1.394 (PMC7810407; doi:10.20411/pai.v5i1.394)
Supplement: Supplementary Figure 4 [file pai-5-382-s06.pdf]

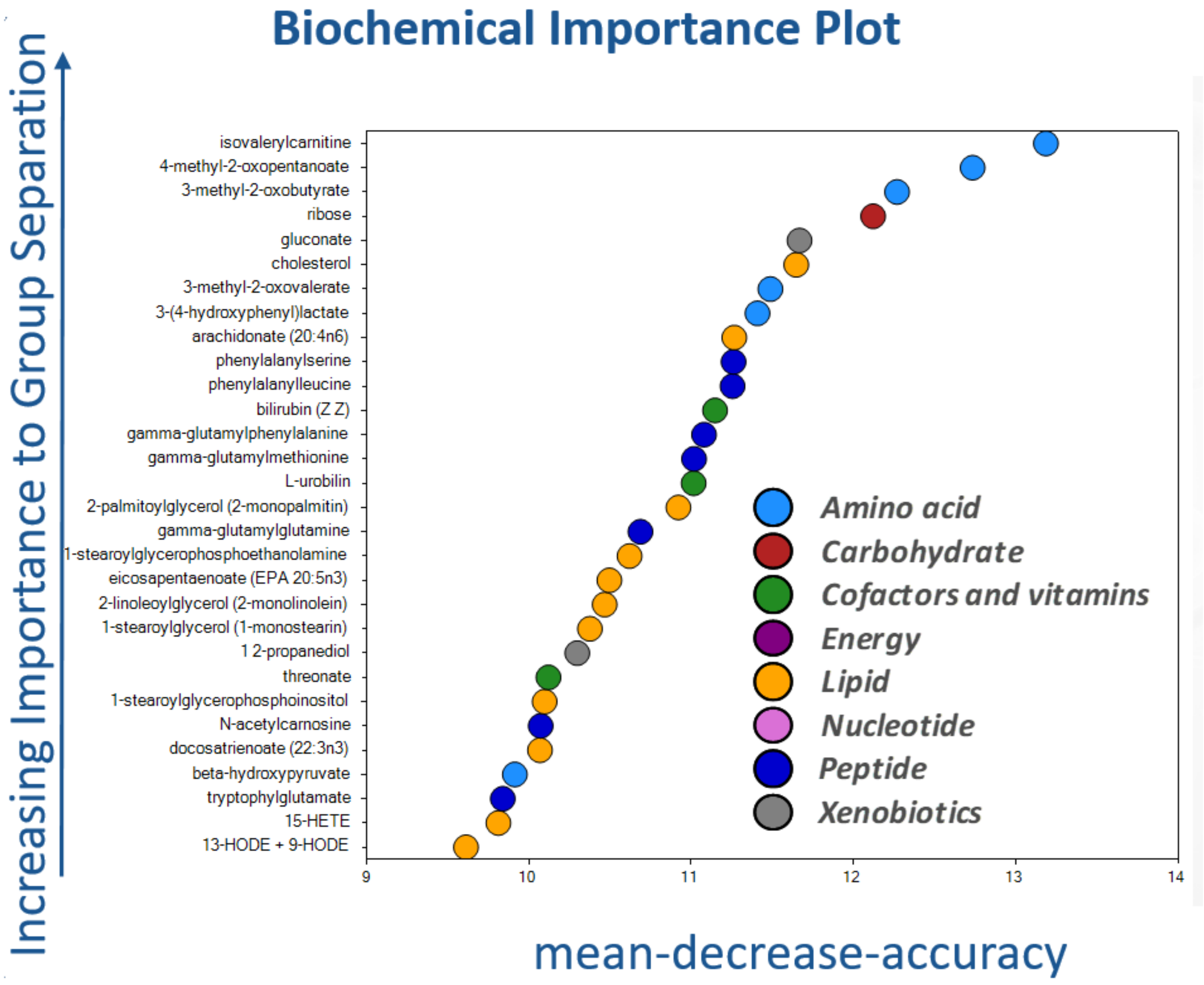

Figure S4 — Biochemical Importance Plot

This is an output of the random forest algorithm. The most important chemicals are listed from top to bottom on the y-axis as most to least important. Mean decrease in accuracy measures how the model performs without a given metabolite. A higher value denotes the importance of that metabolite in predicting the group assignment (control vs antibiotic).
