# Supplementary material for: Changes in the Serum Metabolome of Patients Treated With Broad-Spectrum Antibiotics
Source: Pathog Immun. 2020 Dec 29;5(1):382–418. doi: 10.20411/pai.v5i1.394 (PMC7810407; doi:10.20411/pai.v5i1.394)
Supplement: Supplementary Table 1 [file pai-5-382-s07.pdf]

Statistical Comparisons					
ANOVA Contrasts		Total biochemicals $P \leq 0.05$	Biochemicals (↑↓)	Total biochemicals $0.05 < P < 0.10$	Biochemicals (↑↓)
Abx Treated* / *Control	***Pre-Tx***	39	15|24	48	28|20
	Mid-Tx	37	6|31	25	7|18
	Post-Tx	54	37|17	40	23|17
Control Across Time	***Mid-Tx* / *Pre-Tx***	13	1|12	10	5|5
	Post-Tx* / *Pre-Tx	69	9|60	20	3|17
	Post-Tx* / *Mid-Tx	a	4|33	29	4|25
ABX Treated Across Time	***Mid-Tx* / *Pre-Tx***	82	24|58	47	10|37
	Post-Tx* / *Pre-Tx	153	37|116	36	14|22
	Post-Tx* / *Mid-Tx	45	8|37	30	14|16

Table S1: Aggregate data for all 499 chemicals as well as 5 controls and 20 antibiotic-treated individuals subjected to an ANOVA with treatment x time.
